# Impact of educational program on knowledge, attitude and preventive behaviors related to HIV STIs in female sex workers in Shiraz south Iran

**DOI:** 10.1186/1742-4690-9-S1-P97

**Published:** 2012-05-25

**Authors:** Mahmood Amini Lari, Minoo Ali pour Sakha, Farbod Ebadi Far Azra, Parvin Afsar Kazerooni, Mehrab Sayadi

**Affiliations:** 1 Shiraz HIV/AIDS Research Center, Shiraz, Israel

## Background

Female sex workers (FSW) have been well identified as core groups that play an important role in the effluence of sexually transmitted diseases (STD) and HIV. The purpose of present study was to assess the impact of educational intervention on promoting STIs /HIV related knowledge, attitude and preventive behaviors among female sex workers In Shiraz Iran in 2009.

## Methods

In this quasi-experimental study with a pre-test -post-test design which was conducted from February to July 2009, 80 female sex workers were selected from 5 Shiraz Drop in Centers using the classified random sampling technique. At the beginning FSWs´ HIV/STI related information was assessed in a safe place by interview, using standard questioners as pre-test and then educational intervention program (lecture, face to face education, pamphlet, educational movie, role playing health educational competition) have performed. After 2 months post test was administered to evaluate the effect of interventional program.

## Results

The average age of the participants was 32.62± 9.11 and the average number of participants´ partners was 4 (range 1-15).After the educational intervention, there was a significant difference between the pre-test and post-test knowledge scores ,it means that the mean score of general knowledge related to HIV/STI increased from 13.7±0.95 to 19.47±11.62 (P < 0.01). Similarly, there was a significant improvement in the sex workers´ attitude and their sexual preventive behaviors such as safe sex and condom use (P < 0.01).

## Conclusion

According to results of this study, the educational program was successful in increasing and promoting the HIV/AIDS-related knowledge and attitudes of the participants and enhancing their HIV sexual risk behaviors. More interventional studies should be developed for other high risk population.

